# Obesity-driven inflammation and immune dysregulation in cancer development and progression

**DOI:** 10.3389/fonc.2026.1947958

**Published:** 2026-10-12

**Authors:** Pritika Pandey, Jeb F. Struder, Erika T. Rezeli, Evan M. Paules, Stephen D. Hursting, Michael F. Coleman, Kristin A. Altwegg

**Affiliations:** 1 Department of Nutrition, University of North Carolina at Chapel Hill, Chapel Hill, NC, United States; 2 Department of Exercise and Sport Science, University of North Carolina at Chapel Hill, Chapel Hill, NC, United States; 3 Nutrition Research Institute, University of North Carolina at Chapel Hill, Kannapolis, NC, United States; 4 Lineberger Comprehensive Cancer Center, University of North Carolina at Chapel Hill, Chapel Hill, NC, United States

**Keywords:** adipokines, cancer prevention, immune checkpoint inhibition, immunometabolism, inflammation, insulin resistance, macrophages, myeloid-derived suppressor cells

## Abstract

Obesity increases the risk and progression of at least 13 types of cancer. Although the epidemiologic links between excess adiposity and these cancers are well documented, the biological mechanisms linking obesity to carcinogenesis and tumor progression remain incompletely understood. White adipose tissue in obesity is a source of inflammatory and immunosuppressive signals that act systemically, driving metabolic, inflammatory, and immune dysfunction whose consequences differ across tumor types. As adipose tissue expands and adipocytes enlarge, the blood supply becomes inadequate, leading to hypoxia, fibrosis, lipolytic stress, macrophage recruitment, and chronic low-grade inflammation. These obesity-induced perturbations generate endocrine, cytokine, lipid, and metabolic signals that act systemically, within the tumor microenvironment (TME), and/or cell-intrinsically to activate oncogenic and tumor-promoting pathways. The same signals reshape innate and adaptive immunity, promoting tumor-permissive macrophage states and myeloid-derived suppressor cell expansion, while the effector arms weaken as antigen presentation, natural killer cell and T cell function all decline. This review synthesizes current evidence linking obesity-associated inflammation and immune dysregulation to cancer initiation, progression, and treatment responses across multiple obesity-related cancers. Emphasis is placed on adipose tissue inflammation, myeloid cell biology, T cell immunometabolism, endocrine-inflammatory crosstalk, and the relationship between obesity and immunotherapy. Finally, we discuss translational opportunities for preventing or intercepting obesity-related cancers, including weight loss achieved through lifestyle, surgical, or pharmacologic approaches, with an emphasis on GLP-1 receptor agonists given their substantial efficacy, rapid clinical uptake, and potential to reshape obesity-related cancer prevention and survivorship strategies. Defining the metabolic, immune, and inflammatory phenotypes that drive obesity-associated cancer risk and progression will be essential for developing more precise, mechanism-based strategies for prevention, treatment, and survivorship care.

## 1 Introduction

Obesity increases the risk of at least 13 cancer types and is a major modifiable driver of the global cancer burden (1, 2). Cancers most strongly linked to obesity include postmenopausal breast, endometrial, colorectal, liver, pancreatic, renal cell, esophageal, gastric cardia, gallbladder, ovarian, thyroid, meningioma, and multiple myeloma (1). There is also evidence linking obesity to bladder cancer, aggressive prostate cancer, diffuse large B-cell lymphoma, non-Hodgkin lymphoma, glioma, and certain leukemias (2). The risk of obesity-associated cancer differs for each cancer type and depends on factors such as sex, menopausal status, age, body fat distribution, metabolic health, tumor subtype, ancestry, environmental exposures, and treatment setting (3). These findings argue against a unifying mechanism explaining all the known obesity-cancer links and indicate instead a network of tissue-specific processes driven by systemic metabolic stress.

Visceral and subcutaneous white adipose tissue function together as an endocrine, immune, and metabolic organ, while brown adipose tissue serves as a specialized thermogenic and secretory organ that dissipates energy as heat (4). In the early stages of excess energy intake, adipose tissue expands in a relatively healthy fashion, stores excess lipid, maintains insulin sensitivity, and remodels its vasculature and extracellular matrix. With progressive adipocyte hypertrophy, adipose tissue becomes hypoxic, fibrotic, inflamed, and insulin resistant (4). Macrophages and other immune cells are attracted, and adipocytes change their secretome. This results in the release of inflammatory cytokines, lipids, and extracellular vesicles into local tissue and circulation, contributing to further adipose dysregulation, adipocyte lysis, and inflammation (4). Unlike acute inflammation caused by sudden injury, infection, allergic reactions, or chemical exposures, obesity-associated inflammation is usually low-grade, tied to nutrient overload, and unresolved (5). Over time, this inflammatory state can alter several hallmarks of cancer, including epithelial cell turnover, oxidative stress, DNA damage, epigenetic changes, stem and progenitor cell expansion, angiogenesis, and resistance to apoptosis (6). Once tumors are established, the same obesity-related perturbations support immune escape, invasion, metastasis, and treatment resistance (7).

Obesity also alters the abundance, localization, metabolism, and function of many immune cell populations that reside in adipose tissue and can enter or surround tumors (8). Macrophages and neutrophils often accumulate in expanding adipose tissue, including tumor-adjacent fat where their secretome supports tumor growth, tissue remodeling, myeloid-derived suppressor cell expansion, and impaired functions of dendritic cells, natural killer (NK) cells, and T cells. Thus, obesity-associated inflammation creates a tumor microenvironment (TME) marked by both tumor-promoting inflammation and impaired immune control, a combination that favors tumor growth, spread, and resistance to therapy.

Many prior mechanistic reviews on obesity and cancer, including our own (5), have focused heavily on systemic metabolic factors, including insulin, insulin-like growth factor (IGF-1), adipokines, and sex steroids, and these remain key contributors. In recent years, however, there has been a broadening appreciation that obesity represents a condition of immunometabolic reprogramming that impacts several different biosystems, including the TME. Rather than seeing obesity as multiple distinct effects such as endocrine signaling, nutrient overload, perturbations of glucose and lipid metabolism, stromal remodeling, and immune cell dysfunction, a growing number of investigators view these biological responses to obesity as connected and interacting with each other. Herein, we focus on inflammatory and immune processes that underlie obesity-associated cancers, starting with adipose tissue dysfunction as a systemic inflammatory and immunomodulatory platform, followed by a discussion of cytokine, adipokine, lipid, and metabolic pathways that connect obesity to tumor-promoting immune evasion. Further, we review tissue-specific mechanisms across major obesity-associated cancers and consider implications for prevention and therapy. We conclude with translational opportunities and research priorities, including the use of New Approach Methodologies (NAMs) increasingly encouraged by NIH and other agencies to complement and, in some contexts, reduce reliance on traditional animal models (9). **Figure 1** summarizes how obesity-associated inflammation and immune dysregulation contribute to cancer development and progression.

**Figure 1:**
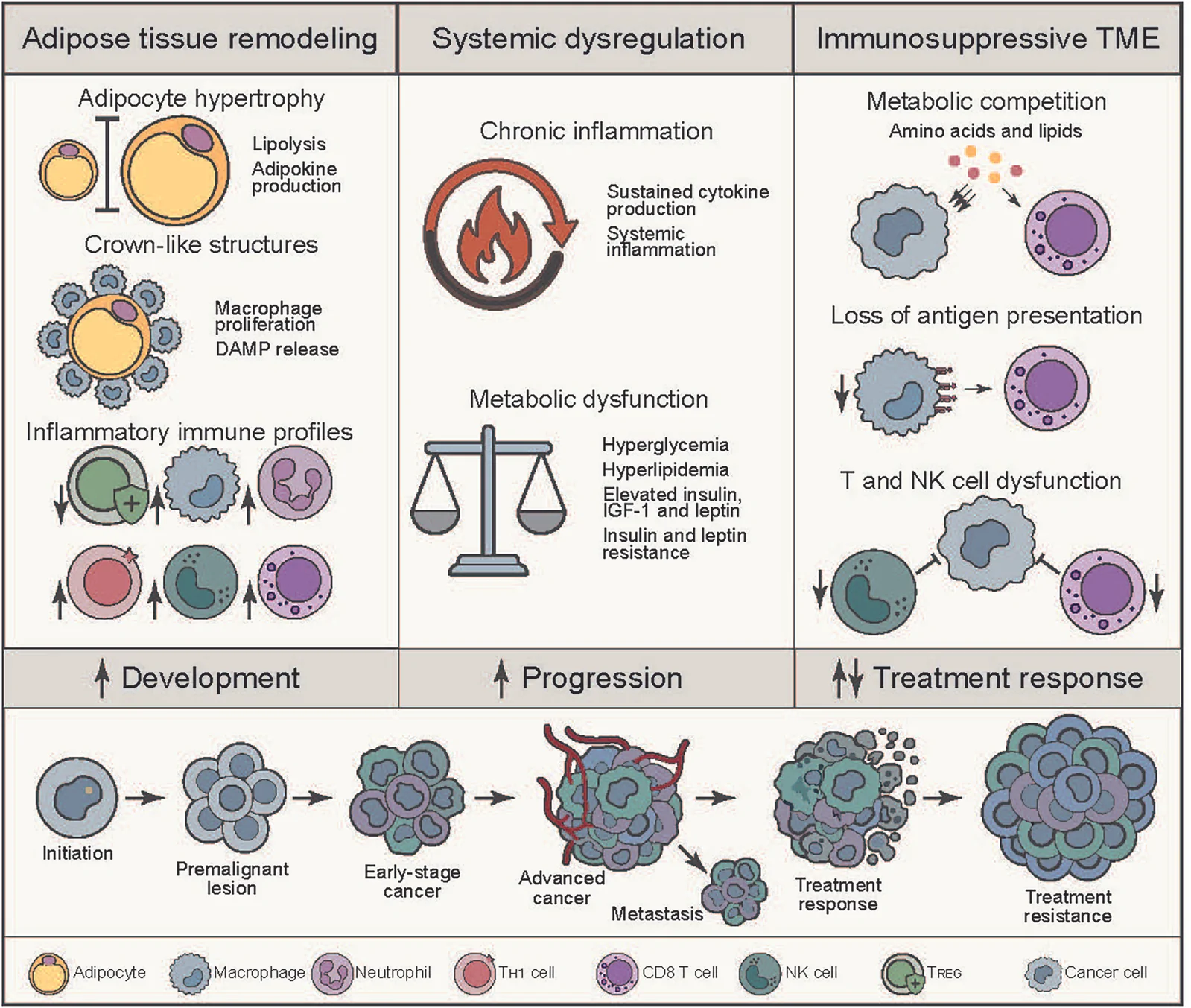
Obesity-associated immunometabolic dysregulation: effects on cancer development and progression. In obesity-driven solid tumors, adipose tissue undergoes remodeling characterized by adipocyte hypertrophy, hypoxia, fibrosis, immune cell infiltration, with altered secretion of adipokines, cytokines, and lipids. These changes contribute to systemic metabolic dysfunction, chronic inflammation, and the development of an immunosuppressive tumor microenvironment marked by dysfunctional antitumor immunity and increased accumulation of suppressive immune populations. Collectively, these processes support cancer initiation, progression, metastatic dissemination, and resistance or altered responsiveness to therapeutic interventions, including immunotherapy. While most of the research to date on this topic has focused on solid tumors, similar inflammatory and immune mechanisms also operate within the bone marrow microenvironment to alter the hematopoietic niche and contribute to hematologic malignancies. These bone marrow changes can promote dysregulated myelopoiesis and clonal hematopoiesis, suppress antigen presentation and T and natural killer cell activity, and provide metabolic and prosurvival support to malignant hematopoietic cells. Collectively, systemic and bone marrow–specific effects of obesity may facilitate the initiation and progression of hematologic malignancies, protect malignant cells from therapy, and alter responses to chemotherapy, targeted agents, and immunotherapy.

## 2 Adipose tissue dysfunction as an immunometabolic driver of cancer risk

Adipose tissue is increasingly recognized as a highly dynamic endocrine and immune organ, with multiple functions beyond energy storage. It is also remarkably heterogeneous, with distinct depots differing in developmental origin, cellular composition, endocrine function, and inflammatory potential (10). Generally, visceral adipose tissue exhibits increased immune cell infiltration, fibrosis, insulin resistance, and pro-inflammatory cytokine production, whereas subcutaneous depots retain greater metabolic flexibility before progressing toward inflammation (11, 12). In addition, emerging evidence indicates that dietary composition, independent of adiposity, can reshape adipose immune programs and immunometabolic signaling (13).

The shift from healthy to pathologic adipose expansion is a key early event in obesity-related disease. Healthy expansion involves coordinated angiogenesis, extracellular matrix remodeling, adipocyte hyperplasia, efficient lipid storage, and preserved insulin sensitivity (11). This remarkable plasticity enables adipose tissue to accommodate excess energy while minimizing metabolic stress and inflammation. With chronic nutrient excess, however, adipose tissue hypertrophy can outpace angiogenesis, resulting in inadequate oxygen delivery and hypoxia. This hypoxic environment promotes adipocyte stress and cell death, pathologic extracellular matrix remodeling and fibrosis, and the recruitment of inflammatory immune cells, initiating a self-reinforcing cycle of adipose tissue dysfunction and inflammation (12).

Hypertrophic adipocytes show higher rates of basal lipolysis, altered adipokine production, mitochondrial and endoplasmic reticulum stress, and release of danger-associated molecular patterns (DAMPs), leading to innate immune activation (12, 14). Increasingly stressed, dysfunctional adipocytes can also lead to necrotic or, under some inflammatory conditions, pyroptotic cell death that attracts circulating monocytes to infiltrate and differentiate into adipose tissue macrophages (15, 16). These macrophages surround dead or dying adipocytes, forming characteristic crown-like structures (CLS) (17). In CLS, these lipid-laden macrophages produce proinflammatory factors, reactive oxygen species, and matrix-remodeling enzymes, sustaining chronic low-grade inflammation (12, 14, 17).

Obesity-associated adipose dysfunction promotes cancer via local tissue effects and systemic signaling. Local effects often include inflamed adipose depots that directly border epithelial tissue or tumors, such as mammary adipose tissue in breast cancer, pericolonic and mesenteric fat in colorectal cancer, peripancreatic adipose tissue in pancreatic cancer, omental fat in ovarian cancer, and perinephric fat in renal cell carcinoma (10, 18, 19). In these adipose microenvironments, adipocytes and infiltrating immune cells communicate with epithelial, stromal, and tumor cells in ways that impact extracellular matrix remodeling, immune function, and tumor progression (18–20).

Systemically, obesity-associated adipose dysfunction alters endocrine and inflammatory signaling, resulting in hyperinsulinemia, dyslipidemia, and adipokine imbalance. In addition, elevated circulating inflammatory mediators, including interleukin-1 beta (IL-1β), interleukin-6 (IL-6), and tumor necrosis factor-alpha (TNF-α), influence epithelial proliferation, immune cell differentiation, stromal remodeling, and tumor metabolism in distant tissues (10, 21). Taken together, these findings position dysfunctional adipose tissue as a mechanistic nexus through which chronic nutrient excess is translated into local and systemic inflammatory, metabolic, and endocrine signals that promote cancer initiation and sustain tumor progression across multiple organ systems.

## 3 Cytokine and inflammatory signaling networks

IL-6 is a central mediator linking obesity-associated inflammation to tumor-promoting signaling. Circulating IL-6 levels rise with obesity and adipose tissue dysfunction, where it functions as a major adipocytokine (22, 23). Adipose tissue macrophages, stromal cells, as well as many tumor cells produce IL-6, which signals through the IL-6 receptor and glycoprotein 130 (gp130) to activate janus kinase/signal transducers and activators of transcription 3 (JAK/STAT3) signaling (24). Persistent STAT3 activation promotes multiple aspects of cancer biology such as cell survival, proliferation, angiogenesis, invasion, stemness, and immune evasion (25). Beyond its actions on tumor cells, IL-6 reduces antitumor immunity by inhibiting dendritic cell function, promoting immunosuppressive myeloid cell programs, and affecting T cell differentiation (26–28). In a landmark study, Park et al. indicated that obesity-induced elevations of IL-6 and TNF-α promote hepatocellular carcinoma by activating STAT3 signaling (29). In addition, IL-6 promotes cancer progression through both shared STAT3-dependent pathways and tissue-specific mechanisms (30).

Across multiple cancer types, IL-6 signaling has been implicated in processes that support tumor growth, survival, invasion, and metastasis. In breast cancer, IL-6 derived from adipose stromal cells enhances tumor cell migration and invasion (31), while obesity-associated increases in systemic IL-6 can further promote local estrogen production through cyclooxygenase-2 (COX-2)/prostaglandin E2 (PGE2)-dependent aromatase expression in breast preadipocytes (32). Also, IL-6/STAT3 signaling supports intestinal epithelial cell survival and proliferation, thus contributing to colitis-associated tumorigenesis in colorectal cancer (33). Similarly, myeloid cell-derived IL-6 trans-signaling activates the signal transducer and activator of transcription 3/suppressor of cytokine signaling 3 (STAT3/SOCS3) pathway, driving the progression of pancreatic intraepithelial neoplasia and pancreatic tumor development (34). In endometrial cancer, IL-6 promotes tumor cell migration and invasion through STAT3-mediated upregulation of matrix metallopeptidase 2 (MMP2) (35). Collectively, these findings highlight the diverse yet convergent roles of IL-6 in creating a pro-tumorigenic environment across multiple obesity-associated cancers.

TNF-α is another key pro-inflammatory cytokine that links obesity-associated adipose dysfunction to chronic nuclear factor kappa-light-chain-enhancer of activated B cells (NF-κB) activation and tumor-promoting inflammation. TNF-α expression is strongly upregulated in hypertrophic adipose tissue and is largely produced by adipose tissue macrophages but may also be made by adipocytes and other stromal cells (36, 37). Chronic TNF-α signaling promotes insulin resistance, endothelial activation, and persistent NF-κB activation, which sustains obesity-associated inflammation (36, 38). Prolonged activation of NF-κB frequently contributes to tumor-cell survival by stimulating pro-inflammatory cytokines, chemokines, anti-apoptotic proteins, matrix-remodeling enzymes, COX-2, and pro-angiogenic mediators (36, 38). This feed-forward inflammatory circuit supports tumor initiation, progression, invasion, metastasis, and therapeutic resistance, particularly in tissues chronically exposed to obesity-associated metabolic stress (39).

Although the TNF-α/NF-κB axis is an attractive therapeutic target, its effects are highly dependent on cellular and tissue context. Chronic NF-κB activation in inflammatory and malignant cells can sustain tumor-promoting cytokine signaling and cell survival, whereas NF-κB activity in normal epithelial compartments can protect against tissue injury (39, 40). Thus, broad pathway inhibition may disrupt protective functions alongside tumor-promoting signaling, favoring more selective approaches that target specific cellular compartments or downstream effectors (39).

Obesity also activates innate immune signaling via the NLR family pyrin domain-containing 3 (NLRP3) inflammasome, a multiprotein complex that responds to cellular and metabolic stress signals, including saturated fatty acids, cholesterol crystals, and extracellular ATP (41). Obesity-associated lipotoxicity, tissue hypoxia, and mitochondrial stress provide signals that prime or activate the NLRP3 inflammasome in adipose tissue macrophages, resulting in caspase-1-dependent processing of pro-IL-1β and pro-IL-18 into their mature forms (41, 42). IL-1β increases leukocyte trafficking, endothelial activation, angiogenesis, and expansion of immunosuppressive myeloid populations that contribute to a pro-tumorigenic microenvironment (43).

The role of inflammasome signaling in cancer is highly context dependent. In the liver, NLRP3/IL-1β signaling promotes inflammation, hepatic stellate-cell activation, and fibrosis, all of which contribute to establishing a tissue environment associated with hepatocarcinogenesis (44, 45). In colorectal cancer, by contrast, inflammasome signaling can have both tumor-promoting and tumor-suppressive effects depending on the cellular context and stage of disease (44, 46). The randomized, double-blind, placebo-controlled CANTOS trial provided clinical proof of concept by showing that IL-1β blockade with canakinumab was associated with lower lung cancer incidence and mortality (47). Although these exploratory findings support the possibility that targeting systemic inflammation can influence cancer development, the trial was conducted in a cardiovascular population and was not designed to establish cancer-preventive efficacy or the underlying tumor-specific mechanisms (47, 48).

Cyclooxygenase-2 (COX-2)/prostaglandin E2 (PGE2) is another important inflammatory pathway linking obesity-associated signaling to cancer. In an obesity-associated breast cancer model, systemic IL-6 increased COX-2 expression and PGE2 production in breast cancer cells, leading to increased aromatase expression in neighboring preadipocytes and increased estrogen receptor-α activity, which promoted breast cancer cell proliferation (32). Eicosanoids mediate interactions between malignant cells and the TME, and COX-2/PGE2 signaling contributes to tumor-cell proliferation and survival, angiogenesis, invasion, immune modulation, and metastasis (49, 50). PGE2 also promotes an immunosuppressive TME through E-prostanoid (EP) receptor signaling, including suppression of cytotoxic T cell and natural killer (NK) cell activity and expansion or recruitment of regulatory T cells and myeloid-derived suppressor cells (49, 50). These effects provide an additional mechanism through which chronic inflammatory signaling can promote colorectal carcinogenesis. Randomized trials of daily aspirin use have demonstrated reduced colorectal cancer incidence and mortality, consistent with inhibiting COX-2/PGE2 signaling (51). However, aspirin chemoprevention is unlikely to benefit all individuals equally. Observational studies suggest that the effects of aspirin may vary according to tumor biology, including COX-2 expression (52) and phosphatidylinositol-4,5-bisphosphate 3-kinase catalytic subunit alpha (PIK3CA) mutation status (53), underscoring the need for additional studies to identify patient populations most likely to derive benefit while minimizing harm (54, 55).

In hematologic malignancies, unlike solid tumors, malignant cells arise within and circulate through hematopoietic and immune compartments. Consequently, obesity may influence these cancers not only through systemic metabolic and inflammatory signals but also by remodeling the bone marrow niches that regulate hematopoiesis, immune function, and malignant-cell survival (56). Bone marrow adipose tissue is a metabolically active component of these niches. Marrow adipocytes produce adipokines, cytokines, lipids, and other metabolites that can affect hematopoietic stem and progenitor cells and neighboring malignant cells (56, 57). Obesity-associated elevations in insulin/IGF signaling, the leptin: adiponectin ratio, free fatty acids, IL-1β, IL-6, TNF-α, and IL-17A may promote inflammatory and myeloid-biased hematopoiesis (56, 57). In preclinical models, IL-6-rich fatty marrow favors expansion of DNA methyltransferase (DNMT) 3A-mutant clones, while obesity-induced inflammatory signaling accelerates protein tyrosine phosphatase (PTPN)11-mutant myeloid disease (58, 59). These findings suggest that obesity may facilitate early clonal selection as well as progression of established malignancy.

Interactions between adipocytes and malignant cells are reciprocal. Myeloma cells stimulate adipocyte lipolysis and use released fatty acids to support growth (60), whereas leukemia cells can exploit adipocyte-derived glutamine and other metabolites to resist therapy (61). Adipose and stromal interactions can also promote leukemia cell survival and treatment resistance through several distinct mechanisms. Adipocyte and stromal cell interactions can alter leukemia-cell state and survival signaling (62), while adipose tissue can also provide a protective niche that limits therapeutic efficacy (63). These effects may contribute to minimal residual disease and treatment resistance, consistent with clinical associations between obesity and residual disease in childhood acute lymphoblastic leukemia (ALL) (64).

Obesity additionally remodels marrow immunity by promoting inflammatory macrophages and dysfunctional myelopoiesis while impairing antigen presentation and T and NK cell activity (57). Unlike the relatively discrete immune microenvironment surrounding many solid tumors, malignant, stromal, and immune cells are intermingled within hematopoietic tissues. Studies should therefore assess marrow adiposity, clonal hematopoiesis, cytokine and adipokine profiles, malignant-cell metabolism, and immune composition rather than relying on body mass index alone.

The findings to date indicate that obesity promotes cancer through an interconnected, functionally overlapping, and context-dependent inflammatory network rather than through any single mediator, underscoring the need to identify the dominant and therapeutically actionable signaling nodes within each tumor and host setting while preserving protective immune and tissue functions.

## 4 Immune-cell remodeling in obesity-associated tumorigenesis

Beyond the soluble mediators described above, obesity also reshapes immune cells, altering their number, location, and function across adipose tissue and the TME. Adipose-derived metabolites play a critical role in lymph node metabolism, and adipokines such as leptin and adiponectin regulate lymphatic vascular function (65). Leptin signals through the leptin receptor and STAT3 on lymphatic endothelial cells to support tube formation and proliferation. However, the sustained hyperleptinemia of obesity induces SOCS3 and instead restrains lymphatic endothelial proliferation and tube formation (66). Adiponectin, which generally falls in obesity, has the opposing effect, promoting lymphatic vessel formation and lymphatic circulation (67). The net consequence of the adipokine milieu in obesity is reduced lymphatic integrity and drainage, which can constrain the trafficking of antigen-presenting cells and lymphocytes between adipose tissue, tumors, and draining lymph nodes (68).

Adipose tissue is infiltrated by lymphocytes and can form fat-associated lymphoid clusters (FALCs), capable of developing into germinal centers (69). Physiologically, regulatory T cells and anti-inflammatory macrophages dominate the adipose tissue immune profile, which also includes innate lymphocytes, NK cells, and invariant T cells. This description derives predominantly from murine visceral (epididymal and gonadal) adipose tissue and applies less well to subcutaneous depots, which are consistently less inflamed and less densely infiltrated (70).

Quantitatively, macrophages account for less than 10% of all cells in lean adipose tissue in both mice and humans and expand in obesity to nearly 40% (37). Foxp3+ regulatory T cells are markedly depleted, collapsing from over 30% of CD4 T cells in healthy visceral adipose tissue to roughly 10% in visceral adipose tissue of obese mice (71). These murine visceral adipose tissue estimates should not be directly extrapolated to other depots, since the magnitude of remodeling differs by species and by depot. Obesity remodels the adipose tissue immune profile toward inflammatory subsets, including B cells, CD8+ T cells, and TH1, and suppresses regulatory T cells (69). Many of the prevalent T cell subsets in adipose tissue are resident and directly contribute to its metabolic health, revealing a bidirectional relationship between inflammation and metabolic dysfunction in adipose tissue (72, 73). Collectively, the displacement of the resident populations that normally enforce tolerance and support tissue repair in adipose tissue (regulatory T cells and anti-inflammatory macrophages) by effector and inflammatory subsets whose chronic activation drives tissue damage but fails to generate productive antitumor immunity contributes to adipose tissue dysfunction. Since remodeling exhibits depot specific effects, the immune context a tumor encounters depends on which adipose tissue it encounters. Tumor-infiltrating lymphocytes (TIL) are critical mediators of antitumor immunity and, as in adipose tissue, their function within solid tumors is sharply limited by obesity. Altered adipokine signaling in response to increased systemic levels of leptin and decreased adiponectin with obesity promotes T cell dysfunction (74). Acutely, leptin acts through the leptin receptor to activate JAK2/STAT3 and phosphoinositide 3-kinase (PI3K)/ mammalian target of rapamycin (mTOR), and supports T cell activation, glycolytic reprogramming, and effector differentiation (75). However, sustained elevation of leptin in obesity drives Programmed cell death protein 1 (PD-1) expression and functional impairment, with reduced interferon-gamma (IFN-γ) and TNF-α production and impaired cytotoxicity (74, 76).

Notably, induction of PD-1 in TILs of obese mice is not observed in all models (77). In the solid tumor microenvironment, obesity promotes competition between cancer cells and TILs for lipids, limiting TIL supply and impairing TIL function. This is achieved through reduced expression of prolyl hydroxylase domain 3 (PHD3) in tumor cells, which enables increased uptake of free fatty acids that sustain tumor growth. This accelerated uptake of lipids results in a TME lacking the free fatty acids on which CD8 TIL rely for proliferation (78). Similarly, suppression of amino acid metabolism in TILs contributes to the loss of TIL function and, therefore, accelerated tumor growth. Specifically, the serum concentrations of amino acids are reduced in obese mice, with concordant suppression of intracellular amino acid metabolites and mTOR signaling in TIL from obese mice (77).

Analogously, suppression of metabolic fitness in NK cells curtails their capacity to control tumors (79). Elevation of circulating free fatty acids in obesity results in lipid accumulation and storage in NK cells, driven by activation of a peroxisome proliferator-activated receptor alpha (PPAR-α) and peroxisome proliferator-activated receptor delta (PPAR-δ) transcriptional program. Metabolic and transcriptional programs repress mTORC1 signaling and glycolysis, which are required for expression of granzyme B, perforin, and IFN-γ. Critically, mobilization of lytic granules to the NK-tumor cell immune synapse is impaired. This defect in lipid metabolism can be reversed with inhibitors of PPAR signaling, which results in restored NK-mediated tumor control. While intracellular lipid accumulation in TILs is not increased with obesity (77), mobilization of intracellular lipid droplets does enhance TIL mediated tumor control (80).

Dietary lipid composition also plays a key role in curtailing TIL and NK-mediated tumor control. High-fat diets that induce elevated levels of long-chain acylcarnitines (e.g. butter and lard), but not other lipid sources (e.g. palm oil), result in similar levels of obesity and marked suppression of both TIL and NK function (13). Mechanistically, long-chain acylcarnitines compromise mitochondrial function and thereby limit TIL activation (13). Predictably, declining T and NK cell function with obesity is paralleled by a loss of selection pressure from T cell-mediated immune control, resulting in a marked loss of immunoediting in obesity (81). Collectively, systemic perturbations in lipid metabolism direct metabolic reprogramming that favors tumor cell growth and survival, while enforcing maladaptive responses in TIL and NK cells that prohibit the glycolytic and biosynthetic metabolism required for tumor control.

Turning from lymphocytes to the myeloid compartment, macrophages are the cells most profoundly remodeled by obesity. The origins of adipose tissue macrophages (ATMs) are currently under debate, with evidence supporting both yolk sac and bone marrow-derived ATMs (82). Under normal conditions, ATMs maintain an anti-inflammatory state and contribute to local lipid metabolism, proliferating after high-fat diet feeding (83). Yolk sac-derived ATMs represent a substantial fraction of resident ATMs and express T-cell/transmembrane immunoglobulin and mucin domain containing 4 (TIM-4) (84), and depletion of these TIM4+ resident macrophages impairs adipose tissue function and lipid handling in adulthood (85), emphasizing the importance of resident ATMs for metabolic maintenance. This anti-inflammatory resident ATM program transitions with obesity. Obese adipose tissue harbors a wave of monocyte-derived ATMs, which are more immunosuppressive and less efficient at endocytosis than resident ATMs (86). Among these monocyte-derived populations, lipid-associated macrophages (LAMs) especially originate from monocytes (87) and accumulate extensively within CLS,, where they proliferate.

LAMs are identified by high expression of triggering receptor expressed on myeloid cells 2 (TREM2), a lipid-sensing surface receptor that regulates lipid uptake and metabolism, efferocytosis, and an anti-inflammatory transcriptional program. In adipose tissue LAMs take up lipids released by dying adipocytes, preventing ectopic deposition (88). LAM depletion by targeting TREM2 induces adipocyte hypertrophy, glucose intolerance, systemic inflammation, and hypercholesterolemia (89). However, it remains unclear whether these changes are fully dependent on LAM loss or if TREM2 loss in other tissues, such as liver macrophages in metabolic dysfunction-associated steatohepatitis (MASH), contributes to these metabolic outcomes (90, 91). For example, prolonged hypernutrition impairs TREM2-dependent efferocytosis by liver macrophages, resulting in defective clearance of lipids and dying hepatocytes, sustained inflammation, and ultimately progression of steatosis to steatohepatitis (91, 92). TREM2 loss therefore may carry metabolic consequences well beyond adipose tissue, an important caveat for any attempt to target TREM2 systemically to investigate LAM function in adipose or tumors.

Tumors co-opt the immunosuppressive function of LAMs to dampen antitumor immunity, constituting a potently immunosuppressive myeloid state in the TME (93). TREM2 itself is a potent regulator of antitumor immunity (93), and TREM2 knockout promotes TIL activation and enhances antitumor immunity (94). Mechanistically, LAMs expand within tumors in response to cancer-associated fibroblast (CAF) signaling, driving an immunosuppressive TME (95). Numerous obesity-associated systemic inflammatory cues suppress TAM antigen presentation to TILs via PD-1 signaling in TAMs, forming a key link between innate and adaptive immunosuppression in the obese tumor setting (96). Obesity-associated metabolic and inflammatory cues induce PD-1 on tumor-associated macrophages downstream of mTORC1 signaling and glycolysis, and PD-1 signaling on TAMs in turn further restrains glycolysis, phagocytosis, and antigen presentation (96). Myeloid-restricted deletion of PD-1 or pharmacological blockade slows tumor growth and improves CD8+ T cell function (96). Importantly, a small observational analysis in humans with obesity found that weight loss is sufficient to relieve the obesity-enforced PD-1 expression on TAMs (96).

Beyond lipid metabolism, obesity further sustains an immunosuppressive TME, in part through detection of acidity by GPR65, a proton-sensing G protein-coupled receptor in TAMs, thereby limiting the efficacy of TIL-mediated tumor control (97). In obesity, accumulation of oleic acid promotes acidification of the TME by tumor cells, the rising proton concentration is then sensed by GPR65 on TAMs (97). GPR65 signaling in TAMs promotes immunosuppression, blocking tumor control by TILs, and permitting accelerated tumor growth (97).

Obesity also perturbs hematopoiesis in the bone marrow, expanding hematopoietic stem and progenitor pools and skewing output toward the myeloid lineage. Hematopoietic stem cells are imprinted by obesity, and produce inflammatory adipose tissue macrophages after serial adoptive transfer into lean recipients (98). Dietary supplementation with yeast β-glucan restores antitumor responses in obese mice and reverts immune dysfunction which persists after weight loss, by trained myelopoiesis (99). The emergence of central myeloid programming as a plausible explanation for persistent immune dysfunction after weight loss is under-examined and an intriguing research avenue for obesity-associated cancer.

Viewed across immune compartments, obesity does not produce a single immunologic lesion but establishes coordinated dysfunction characterized by impaired lymphatic trafficking and adipose immune homeostasis, metabolically constrained TIL and NK cell activity, expansion of TREM2-high, PD-1-high, and acid-adapted immunosuppressive macrophages, and durable myeloid reprogramming that may persist after weight loss. The resulting immune state shifts the balance from effective tumor surveillance toward immune tolerance and tumor progression, although its dominant features and reversibility likely vary with dietary composition, adipose depot, and cancer type. Key priorities are therefore to map these immune states spatially and longitudinally in human tumors, determine which abnormalities are reversed by weight loss, and identify persistent lesions that require direct intervention. Such studies could guide rational combinations of weight-loss or dietary interventions with cell-specific metabolic, myeloid, or checkpoint-directed therapies while avoiding disruption of the homeostatic functions of lymphatic vessels and resident macrophages.

## 5 Endocrine and metabolic mediators of immune dysfunction

Obesity increases circulating levels of insulin, bioavailable IGF-1, glucose, leptin, and free fatty acids, each of which can influence antitumor immunity independent of any effects on tumor cells (5). Activation of insulin and IGF-1 receptors promotes protein kinase B (AKT)/ mechanistic target of rapamycin complex 1 (mTORC1) signaling to support the glycolytic reprogramming needed for activated CD8+ T cells. Lack of the insulin receptor results in defective glycolysis, decreased IFN-γ production, and antigen-specific expansion in T cells (100). The fuel for effector T cells is primarily supplied by glucose, whose local concentration therefore determines the maximum potential for cytotoxic activity (101). Leptin, a proinflammatory adipokine, can also polarize TAMs and reprogram CD8+ T cell metabolism (102, 103). The mitochondrial, memory arm of the CD8+ T cell response is supported by fatty acid oxidation (104). Glucose-consuming tumors use up local glucose, depriving TILs leading to exhaustion, which is characterized by high PD-1 (105).

Insulin resistance affects T cell cytotoxicity, and insulin can induce tumor programmed death-ligand 1 (PD-L1) to promote immune evasion (106). Persistent hyperleptinemia drives PD-1-mediated CD8+ T cell exhaustion through STAT3, lowering IFN-γ and TNF-α (74) and producing metabolically impaired TIL (76). In the lipid-rich obese niche, tumor cells outcompete T cells for fatty acids (78), while long-chain species accumulate and impair mitochondrial function (13), and NK cells undergo PPAR-driven lipid paralysis that blunts cytotoxicity (79).

In sum, obesity converts endocrine signals and nutrients normally required for immune activation and memory into chronic metabolic pressures that promote PD-1/PD-L1 signaling, terminal T cell exhaustion, NK cell lipid paralysis, and immunosuppressive macrophage polarization. A central research priority is to determine how systemic hyperinsulinemia and hyperleptinemia interact with local competition for glucose, fatty acids, and amino acids, and which resulting immune defects remain reversible. Longitudinal studies should combine metabolic and immune phenotyping with interventions such as weight loss, dietary modification, or selective modulation of insulin/IGF-1, leptin–STAT3, mTOR, and lipid–PPAR signaling to identify responsive patients and restore antitumor immunity without disrupting the metabolic programs required for normal effector and memory cell function.

## 6 Tissue-specific patterns across obesity-associated solid cancers

A key unifying theme across obesity-associated solid cancers is that excessive adiposity promotes chronic inflammation and immune dysfunction, which impairs antitumor immune surveillance and fosters tumor-supportive stromal, metabolic, and endocrine signaling. However, many of these shared obesity-driven mechanisms manifest differently across cancer types and tissue environments (18–20).

In postmenopausal estrogen receptor positive (ER+) breast cancer, obesity-associated inflammation in breast adipose tissue promotes crown-like structures of the breast (CLS-B), increasing COX-2, PGE2, and aromatase expression (107). The inflammatory signals and adipose-derived estradiol combine to suppress antitumor immunity and promote ER+ breast cancer by reducing cytotoxic T cell infiltration and promoting immune-tolerant macrophage polarization (107). In triple-negative breast cancer, the pro-cancer effects of obesity are estrogen-independent and involve immunosuppressive inflammatory cytokine/chemokine production (such as CXCL1/2, which stimulates myeloid-derived suppressor cell recruitment, and IL-6, which drives STAT3 signaling). Moreover, increased insulin receptor signaling and adipocyte–tumor metabolic crosstalk (such as increased leptin signaling) depletes CD8+ T cell energy substrates and decreases effector function (108).

In endometrial cancer, unopposed estrogen remains a central driver of tumorigenesis, but obesity also promotes a pro-inflammatory, metabolically dysregulated, and immunologically altered endometrial microenvironment. Insulin resistance, hyperinsulinemia, adipokine dysregulation, macrophage infiltration, and impaired immune surveillance can facilitate progression from hyperplasia to carcinoma and modify responses to immunotherapy. Mismatch repair-deficient tumors and those with high neoantigen burdens are intrinsically more immunogenic and therefore more likely to respond to PD-1 blockade (109). However, effective and durable responses depend on immune-cell trafficking and metabolic fitness. Obesity-associated hyperinsulinemia, hyperleptinemia, nutrient competition, and suppressive myeloid signaling may increase PD-1-dependent T cell exhaustion while simultaneously limiting complete or sustained immune reinvigoration (109). Thus, tumor genomic features establish the potential for immune checkpoint inhibitor (ICI) sensitivity, whereas the host metabolic and immune environment may modulate the magnitude and durability of that response.

In colorectal cancer, visceral adipose inflammation increases portal delivery of inflammatory cytokines and free fatty acids to the liver. This promotes hepatic insulin resistance, dysregulates bile acid metabolism (including secondary bile acids that promote colonocyte proliferation) and promotes microbial dysbiosis (110). Moreover, impaired intestinal barrier function allows bacterial translocation that can activate innate immune signaling in the mucosa. Together, these disruptions in the gut–liver axis can promote adenoma progression and tumor growth (110).

Hepatocellular carcinoma (HCC) provides perhaps the clearest example of obesity-driven inflammation promoting carcinogenesis. Metabolic dysfunction-associated steatohepatitis (MASH) induces lipotoxic hepatocyte injury. Specifically, excess free fatty acids and associated toxic metabolites induce hepatic endoplasmic reticulum stress, mitochondrial dysfunction and caspase-dependent apoptosis, resulting in hepatocyte cell death and release of DAMPs that activate Kupffer cells via Toll-like receptor-4 and NLRP3. This stimulates IL-1 and TNF-α production and drives a permissive microenvironment for HCC initiation through increased Wnt and IL-6/STAT3 signaling (111).

In pancreatic cancer, obesity intensifies an already desmoplastic and immunosuppressive TME by promoting visceral and intrapancreatic adiposity and adipocyte infiltration into the tumor stroma. Hypertrophic adipocytes provide excess free fatty acids and secrete inflammatory mediators, including IL-1β, which recruits tumor-associated neutrophils that activate pancreatic stellate cells. Activated stellate cells increase collagen and extracellular matrix deposition, producing a dense desmoplastic stroma that compresses blood vessels, exacerbates hypoxia, and creates a physical barrier to cytotoxic T cell infiltration. Together with the accumulation of immunosuppressive myeloid cells, these changes impair immune control while promoting pancreatic tumor progression and treatment resistance (112).

In esophageal adenocarcinoma and gastric cardia cancer, central adiposity similarly acts through both local and systemic mechanisms. Visceral adiposity increases reflux-related injury and the risk of Barrett esophagus while also generating inflammatory cytokines, adipokine imbalance, insulin resistance, and other metabolic signals that sustain mucosal inflammation and may promote progression from metaplasia to neoplasia (113).

In renal cell carcinoma (RCC), obesity-related metabolic dysregulation, oxidative stress, hypertension, and hypoxia may promote tumor initiation and progression, particularly in clear cell RCC (ccRCC). Paradoxically, higher body mass index (BMI) is frequently associated with improved survival after diagnosis. Sanchez et al. (114) found that ccRCC tumors from patients with obesity exhibited greater angiogenic and metabolic signaling but not increased intratumoral inflammation or immune infiltration. Peritumoral adipose tissue from patients with obesity also showed greater hypoxia, inflammation, and immune infiltration, suggesting that the adjacent adipose compartment may influence treatment response. Nevertheless, the survival advantage is less consistent for objective response or progression-free survival and may partly reflect tumor subtype, treatment selection, preserved muscle and nutritional reserves, or reverse causation from cancer-associated weight loss. Thus, the RCC obesity paradox likely reflects a combination of distinct tumor biology and limitations of BMI rather than a protective effect of obesity itself, highlighting the need to integrate weight trajectories, body composition, and tumor and peritumoral molecular profiles (115).

Other obesity-associated solid tumors also show a broad pattern of systemic inflammation and immune dysfunction acting through tissue-specific mechanisms (116, 117). In ovarian cancer, omental adipocyte-tumor interactions, inflammatory and adipokine signaling, and immunosuppressive cells may support tumor growth. In gallbladder cancer, gallstones, altered bile acid metabolism, chronic inflammation, and metabolic dysfunction contribute to carcinogenesis, whereas thyroid cancer may be influenced by insulin resistance, inflammatory signaling, and thyroid-stimulating hormone. Hormonal, metabolic, and inflammatory pathways may similarly contribute to meningioma, while in prostate cancer, obesity-associated inflammation, impaired immune surveillance, and metabolic dysfunction appear more strongly associated with aggressive disease, recurrence, and mortality than with overall incidence (116, 117).

Taken together, obesity establishes a shared systemic substrate of adipose tissue dysfunction, chronic inflammation, endocrine and metabolic dysregulation, and impaired immune surveillance that interacts with each organ’s distinct epithelial vulnerabilities, stromal architecture, hormonal milieu, and microbial or environmental exposures. Comparative and longitudinal studies should determine which components of this biology are conserved across cancers, which are tissue specific, and which can be reversed through lifestyle, metabolic, or anti-inflammatory interventions. Integrating body composition and circulating biomarkers with spatial profiling of adipose and TMEs could identify high-risk phenotypes, explain differences in disease progression and therapeutic response, and guide organ-specific strategies for prevention and interception of obesity-driven solid tumors.

## 7 Tissue-specific patterns across obesity-associated hematologic malignancies

Excess body fatness is an established multiple myeloma risk factor, and obesity may also be associated with monoclonal gammopathy of undetermined significance (MGUS, a premalignant precursor lesion for multiple myeloma), although evidence regarding progression from MGUS to myeloma remains less consistent (1, 2, 118). Associations with other hematologic malignancies are more heterogeneous (1, 2, 119, 120). In childhood ALL, obesity has also been associated in some cohorts with greater minimal residual disease and relapse risk, potentially reflecting altered pharmacokinetics and adipocyte-mediated protection of leukemia cells (61, 64).

Evidence for lymphoma likewise varies by subtype. Large pooled analyses most consistently support an association with diffuse large B-cell lymphoma, particularly when obesity develops earlier in adulthood, whereas associations with follicular lymphoma and several other subtypes are weaker or absent (119, 120). Hematologic malignancies should therefore not be treated as a single outcome: obesity effects likely depend on lineage, developmental stage, tissue niche, treatment regimen, and metabolic phenotype.

Obesity-associated inflammation and immune dysregulation in hematologic cancers operate within the tissues responsible for hematopoiesis and immune cell development. Obesity expands and remodels bone marrow adipose tissue, alters mesenchymal and hematopoietic stem-cell niches, and increases systemic and local concentrations of inflammatory cytokines, adipokines, lipids, and metabolic substrates (121–123). Chronic signaling through IL-1β, IL-6, TNF-α, leptin, and related pathways can promote myeloid-biased hematopoiesis, impair lymphoid and NK cell function, and favor the expansion or persistence of premalignant and malignant hematopoietic clones (121, 122). Emerging evidence also links obesity-associated inflammation with clonal hematopoiesis, suggesting a potential mechanism through which metabolic dysfunction may contribute to the early development of myeloid malignancies (121). However, the causal importance of these pathways in humans and their variation across specific hematologic cancers remain incompletely defined.

Multiple myeloma provides the clearest example of obesity-mediated remodeling of the bone marrow microenvironment. Increased marrow adiposity and altered secretion of IL-6, leptin, adiponectin, insulin-like growth factors, and other stromal mediators may promote plasma-cell survival, proliferation, and resistance to apoptosis (124). Adipocyte-myeloma interactions also support metabolic adaptation, angiogenesis, osteoclast activation, and immune suppression, potentially facilitating progression from MGUS to overt multiple myeloma (123, 124). Reduced adiponectin and increased pro-inflammatory adipokine signaling may be particularly important, although the relative contributions of systemic obesity and local marrow adiposity remain uncertain.

In acute myeloid leukemia, bone marrow adipocytes can be metabolically reprogrammed by leukemic cells to release fatty acids that support fatty acid oxidation, leukemic stem-cell survival, and treatment resistance (123, 125). Obesity-associated inflammation may further alter hematopoietic stem and progenitor-cell function and promote a myeloid-skewed, leukemia-supportive marrow environment. In ALL, adipocytes can provide amino acids, induce quiescence and stress resistance, and sequester or metabolize chemotherapeutic agents, thereby protecting residual leukemia cells from treatment (63, 64). These findings indicate that adipose tissue can function not only as a source of inflammatory signals but also as a metabolic and pharmacologic sanctuary for malignant hematopoietic cells. Obesity-driven systemic metabolic alterations, suppressive myeloid-cell accumulation, and impaired T and NK cell surveillance may similarly promote malignant cell survival and diminish antitumor immunity, although their relative importance likely varies among hematologic malignancy subtypes (56, 57). Unlike most solid tumors, hematologic malignancies arise within and circulate through the same marrow and immune compartments remodeled by obesity, making malignant-cell, stromal, metabolic, and immune effects especially difficult to separate.

Across hematologic malignancies, obesity may transform marrow and systemic immune niches into inflammatory, metabolically permissive, and immunosuppressive environments. Adipocytes and stromal cells provide signals, metabolic fuels, and protection from treatment that can support plasma-cell survival in myeloma, leukemic stem-cell persistence in acute myeloid leukemia (AML), treatment evasion in ALL, and impaired immune surveillance in lymphoid malignancies. Obesity may therefore influence multiple stages of hematologic carcinogenesis, from clonal emergence to progression and therapeutic resistance.

## 8 Obesity and immunotherapy response

Immune checkpoint inhibition (ICI) has become a mainstay for treating numerous cancers. Paradoxically, overweight or obesity has been associated with improved progression-free or overall survival following ICI in several cancer types, including lung, head and neck cancer, and melanoma (126–129), although this association does not extend to all cancers (130). This phenomenon, often termed the “obesity paradox”, may have a biological basis (74, 104). As described above, obesity promotes chronic inflammation, leptin signaling, T cell dysfunction, and increased expression of inhibitory receptors such as PD-1. Although these changes impair endogenous antitumor immunity and can accelerate tumor progression, they may also create a state of checkpoint dependence that is particularly susceptible to PD-1/PD-L1 blockade. In preclinical models, leptin-STAT3 signaling contributes to increased PD-1 expression and dysfunctional CD8+ T cells, which can be preferentially reinvigorated by anti-PD-1 treatment (74, 76). Obesity may therefore simultaneously worsen tumor control in the absence of therapy while increasing the amount of reversible immune suppression available for checkpoint blockade to release.

However, clinical findings are unclear on whether excess adiposity itself causes better ICI response. Most studies are retrospective, and higher BMI may partly identify patients with greater nutritional and metabolic reserves or less pre-existing weight loss and cancer cachexia, both of which are strongly associated with better survival. Conversely, patients classified as normal weight may include individuals who recently lost weight because of aggressive disease. BMI also fails to distinguish adipose tissue from skeletal muscle or to identify sarcopenic obesity, visceral adiposity, or differences in adipose distribution and metabolic health.

Associations may additionally be modified by sex, age, tumor type, disease stage, smoking, performance status, comorbidities, and treatment or dosing patterns. The frequent observation of stronger associations with overall survival than with objective response further suggests that higher BMI may sometimes be a prognostic marker of general health or disease trajectory rather than a specific predictor of ICI sensitivity. Some studies also associate higher BMI with increased immune-related adverse events, potentially reflecting stronger immune activation but complicating interpretation of the apparent benefit.

In RCC, the evidence is variable, with most clinical series reporting higher BMI to be associated with improved survival, although objective response rates are less consistently affected (114, 131). A preclinical model nonetheless found that obesity diminishes PD-1-based responses in renal cancer, indicating that the underlying biology is tumor- and context-dependent and remains incompletely resolved (132). Preclinical studies have also shown that impaired immunoediting in obesity can permit the emergence of more immunogenic tumors that subsequently respond better to ICI treatment (81).

Counterintuitively, although obesity has been associated with improved ICI outcomes in lung cancer, treatment with weight loss-inducing glucagon-like peptide-1 receptor agonists (GLP-1RAs) was associated with further improvement in ICI response (133). This observation argues against interpreting the obesity paradox as evidence that maintaining obesity is beneficial. Instead, obesity may establish an immunologically distinct tumor and host state, whereas subsequent metabolic improvement could reduce inflammatory and metabolic constraints without eliminating tumor immunogenicity or responsiveness to checkpoint blockade. The available GLP-1RA findings are observational, and may be confounded by differences in diabetes control, concurrent medications, healthcare engagement, and patient selection.

A key emerging axis linking obesity with ICI response is alteration of the intestinal microbiota (134–136). The intestinal microbiome can shape systemic immunity and ICI response at distant tumor sites, and some of these effects are transferable through fecal microbiota transplantation (137). Obesity disrupts microbiome composition and microbial metabolism, although the consequences appear context-dependent rather than uniformly detrimental. Recent work suggests that selected obesity-associated microbial communities can enhance ICI responses in preclinical models, and that transfer of these microbiota can restore responsiveness in otherwise resistant recipients (138). Manipulation of dietary fiber represents one highly tractable approach for modifying microbiome-associated metabolites and immune function and could potentially be combined with metabolic or pharmacologic interventions to support durable ICI responses (139).

Overall, the obesity paradox is best understood not as a protective effect of excess adiposity but as a composite host-tumor phenotype in which checkpoint-dependent immune dysfunction, tumor immunogenicity, body composition, metabolic health, and the microbiome can sometimes increase vulnerability to ICI even as obesity promotes tumor progression. Prospective studies should move beyond BMI by incorporating weight trajectory, visceral adiposity, skeletal muscle mass, cachexia, metabolic and inflammatory biomarkers, immune phenotyping, and microbiome composition, ideally with non-ICI comparator groups to distinguish predictive effects from general prognostic associations. These data should guide clinical trials testing whether intentional weight loss, metabolic therapies such as GLP-1RAs, and dietary or microbiome-directed interventions can reduce obesity-related immune dysfunction while preserving or enhancing ICI efficacy and limiting immune-related toxicity.

## 9 Translational strategies for obesity-associated cancer prevention and interception

Unlike many cancer risk factors, obesity and its associated metabolic dysfunction, chronic inflammation, and immune dysregulation are at least partially reversible, creating a unique opportunity for cancer prevention and interception. Observational evidence suggests that intentional weight loss is linked to a lower risk of obesity-related cancers. In the Iowa Women’s Health Study, postmenopausal women who intentionally lost weight had lower rates of overall and obesity-associated cancers (140). Similarly, the Women’s Health Initiative Observational Study found that women who lost at least 5% of their body weight had a significantly reduced risk of obesity-associated cancers, with the strongest association observed for endometrial cancers (141). Additional support comes from studies of bariatric surgery, which have shown that sustained weight loss and improved metabolic health in bariatric surgery patients are associated with lower cancer incidence and mortality. These effects were first reported in the Swedish Obese Subjects study (142) and have been reinforced by more recent analyses, including the SPLENDID study (143). Mechanistic human and preclinical studies provide a plausible framework by which intentional weight loss may reduce obesity-associated cancer risk and progression via reductions in inflammatory cytokines and metabolic hormones and concomitant improvement in CD8+ T cell function (96). Collectively, these findings support targeting obesity as a modifiable biological process that can be targeted for cancer prevention.

Lifestyle intervention remains the cornerstone (144) of obesity-associated cancer prevention because it addresses the underlying metabolic, inflammatory, and immune dysregulation that drives obesity-associated cancer risk. Unfortunately, substantial, sustained weight loss through chronic caloric restriction alone is challenging for most people to maintain. Some do find greater long-term success with intermittent caloric restriction or time-restricted eating relative to chronic restriction (145, 146). Further, food quality has important effects on metabolic health independent of weight change. Dietary patterns emphasizing whole grains, legumes, fruits, vegetables, fiber, and unsaturated fats have been linked to improved insulin sensitivity, reduced systemic inflammation, and favorable changes in the gut microbiome (147). Physical activity complements these effects by reducing visceral adipose inflammation, improving CD8+ T cell trafficking and effector function, modulating adipokine profiles, and preserving skeletal muscle mass (7, 148). Many of these metabolic and immunologic benefits of exercise occur without substantial weight loss, indicating adipose tissue remodeling and metabolic benefits independent of significant weight change (149).

Metabolic pharmacotherapy has become an important option for individuals who are ineligible for bariatric surgery or are unable to achieve or maintain meaningful metabolic improvement through lifestyle change. Metformin generated substantial interest as a possible cancer prevention agent because of its ability to improve insulin sensitivity, lower circulating insulin levels, and suppress mTOR signaling through activation of AMP-activated protein kinase (AMPK) (150). Early observational studies suggested that metformin users experienced lower cancer incidence and better cancer outcomes (150), but enthusiasm waned as randomized clinical trials showed null effects. For example, the MA.32 trial in early-stage breast cancer found no improvement in invasive disease-free survival (151). Interest has now turned toward incretin-based therapies, which activate one or more satiety-related receptors and produce significantly greater weight loss and broader metabolic improvements than achieved with metformin. The GLP-1RA semaglutide and the dual GLP-1RA/glucose-dependent insulinotropic polypeptide receptor (GIPR) agonist tirzepatide produce average weight loss of approximately 15% and 21%, respectively, while substantially reducing visceral, hepatic, epicardial, and other ectopic fat depots (152, 153). Resulting reductions in adiposity are accompanied by improvements in insulin resistance, inflammation, adipokine profiles, and other pathways implicated in obesity-associated carcinogenesis (10).

Given their rapid clinical adoption (144), determining whether GLP-1RAs reduce cancer incidence, improve cancer outcomes, or exert direct tumor-specific effects has become a major research priority, although epidemiologic and clinical data regarding these drugs remain limited and inconclusive. As discussed by Renehan and Pollak, electronic health record (EHR)-based studies, which currently dominate the GLP-1RA epidemiologic literature, are susceptible to several sources of confounding and bias. These include: i) surveillance bias, whereby differences in healthcare contact and cancer screening between GLP-1RA users and comparison groups lead to differential cancer detection; ii) immortal time bias, whereby the period between cohort entry and initiation of GLP-1RA treatment is incorrectly attributed to the treated group, artificially inflating an apparent protective association; iii) confounding by indication and comparator selection, because treatment choice reflects differences in diabetes severity, obesity, comorbidities, and healthcare engagement; and iv) exposure or outcome misclassification and follow-up periods that may be insufficient to capture the long latency of cancer development (154). These limitations make it difficult to determine whether the reported associations reflect true reductions in cancer risk or biases introduced by study design. Consequently, the reported reductions in cancer incidence in patients using these agents should be interpreted cautiously until confirmed in prospective epidemiologic studies and/or randomized clinical trials with cancer-specific endpoints. Well-designed prospective studies should prespecify cancer endpoints, include clinically appropriate active comparator groups with comparable indications and disease severity, provide sufficient follow-up to capture cancer latency, and longitudinally assess treatment exposure, weight change, and body composition beyond BMI.

Preclinical studies provide important biologic support but also have important limitations. Much of the early literature evaluating incretin mimetics used short-term treatment of human tumor xenografts in immunodeficient mice, models that fail to fully capture the metabolic, inflammatory, and immune abnormalities that characterize obesity-associated cancer (155). More recently, studies using immunocompetent models of diet-induced obesity and solid cancers have begun to address these limitations. Our recent work in high fat diet-induced obesity models of mammary, colon and endometrial cancer in immune-intact mice has shown that tirzepatide reverses obesity-driven increases in tumor burden (156–158). The tirzepatide was administered via subcutaneous injection every other day at an escalating dose that achieved and maintained ~20% weight loss in obese mice, mimicking the effects of tirzepatide observed in the SURMOUNT clinical trial (153). These effects occur alongside reductions in adiposity and improvements in insulin sensitivity, systemic inflammation, adipokines, and antitumor immune responses, (assessed by single cell sequencing and spectral flow cytometry) (156–158). What remains as perhaps the most important mechanistic gap in this field is whether the antitumor activity of tirzepatide is simply a consequence of weight loss and improved metabolic health or whether GLP-1/GIP receptor agonism directly influences tumor cells and/or the TME. Our pair-feeding study, in which the comparator group received the same reductions in food intake as observed with tirzepatide-treated mice but were injected with vehicle and not tirzepatide, suggests most of tirzepatide’s anticancer effect is through reduced food intake and associated improvements in body composition and metabolic health (156, 157). Disentangling these mechanisms with experimental approaches such as pair-feeding, incretin receptor knockout studies, and careful evaluation of incretin receptor expression and function in tumor models will be critical for understanding how incretin-based therapies affect cancer development and progression.

The therapeutic potential of GLP-1RAs for reversing the procancer effects of obesity also extends to hematologic malignancies, particularly those linked to an adipocyte-rich bone marrow microenvironment. By reducing adiposity, insulin resistance, systemic inflammation, and potentially bone marrow adiposity, these agents could attenuate the metabolic, inflammatory, and immune signals that support clonal hematopoiesis and malignant-cell survival. Emerging observational findings are intriguing. In a large EHR-based study of patients with type 2 diabetes, GLP-1RA use was associated with lower risks of myelodysplastic syndromes and myeloproliferative neoplasms compared with metformin, and with lower risks of several myeloid, lymphoid, and plasma-cell malignancies compared with insulin (159). In a separate cohort of patients with diabetes and MGUS, GLP-1RA use was associated with a 55% lower risk of progression to multiple myeloma (160). Moreover, in a large retrospective EHR cohort of patients with type 2 diabetes, GLP-1RA use was associated with a 36% lower risk of multiple myeloma, but not AML, chronic myeloid leukemia (CML), or myelodysplastic syndrome (MDS), while SGLT2 inhibitor use did not alter malignancy incidence but was associated with increased mortality in patients with multiple myeloma or AML (161). However, these EHR studies remain vulnerable to confounding by indication, differences in diabetes severity and comparator treatments, and the EHR-related biases described above. Moreover, little is known about whether GLP-1RAs directly affect hematopoietic or malignant cells (161). Mechanistic studies, including in genetically engineered mouse models of hematological malignancies as exemplified by the work of Kapur and colleagues (59), are needed to determine whether these associations reflect weight loss and improved metabolic health, remodeling of the bone marrow adipose and immune microenvironment, direct receptor-mediated effects, or some combination of these mechanisms.

Beyond incretin mimetics, inflammatory and immunometabolic pathways also provide direct therapeutic targets. The CANTOS trial was a randomized, double-blind, placebo-controlled clinical study that established proof of principle by showing reduced lung cancer incidence and mortality following IL-1β blockade (47). These findings transformed oncology research through its demonstration that targeting systemic inflammation may alter cancer development and outcomes (47). Additional candidate targets include IL-6/STAT3, NF-κB, COX-2/PGE2 signaling, inflammasomes, CCL2/CCR2, and CXCL1/2-CXCR2-mediated myeloid recruitment, and metabolic regulators of T cell function and immune evasion (33, 162–165). Safely targeting these pathways could prove challenging, as they also maintain immune homeostasis and tissue repair, so broad or prolonged inhibition may produce unintended consequences favoring context-specific or biomarker-guided approaches.

Taken together, these approaches shift obesity-associated cancer prevention from nonspecific weight reduction toward precision interception of a dynamic, biologically heterogeneous, and partially reversible host state. The central translational objective is to determine which lifestyle, metabolic, and immunomodulatory interventions, alone or in combination, most effectively reverse adipose tissue dysfunction, metabolic dysregulation, chronic inflammation, and immune suppression while preserving skeletal muscle and immune homeostasis. This is especially important for patients experiencing obesity-induced sarcopenia or lean mass loss after receiving pharmacologic agents such as GLP-1RAs (166, 167). Metabolic therapies may also provide a strategy to improve antitumor immunity via improved metabolic health (168). Prospective trials integrating cancer-specific endpoints with longitudinal measures of body composition and metabolic, inflammatory, and immune phenotypes will be essential to distinguish weight-dependent from weight-independent effects, identify the patients most likely to benefit, and define the optimal timing and sequencing of interventions to prevent cancer development or intercept its progression.

## 10 Biomarkers and precision prevention

Although biomarkers have long been used to facilitate therapy selection for patients, there is an ongoing need to identify biomarker signatures that effectively predict responsiveness to preventive or therapeutic interventions. This is particularly the case in treating individuals with obesity, whose dysregulated inflammatory and metabolic profiles make it challenging to match the best therapeutic strategy for a given patient. As mentioned previously, BMI is a crude measure of obesity that can under- and overestimate adiposity, a major driver of obesity-associated metabolic and immune dysfunction (169). This has resulted in new classifications that distinguish individuals with excess adiposity but without organ dysfunction (preclinical obesity) from individuals with organ dysfunction caused by excess adiposity (clinical obesity) (169). These designations have resulted in identifying obesity-related carcinogenesis beginning prior to clinically detectable organ dysfunction, with preclinical obesity being positively associated with 11 cancer types spanning digestive, reproductive, urinary, and endocrine systems (170).

Refining these classifications will depend on markers that capture local and systemic biology. At the tissue level, crown-like structures of the breast (CLS-B) CLS-B mark white adipose inflammation and track with elevated leptin (and leptin:adiponectin ratio), insulin, high-sensitivity C-reactive protein (hsCRP), and IL-6, offering a clinically feasible surrogate of adipose inflammation (20). Spatially resolved transcriptomics further demonstrate that obesity reprograms early breast cancer, diverging from the canonical proliferative and epithelial–mesenchymal transition (EMT) signatures seen in non-obese patients, which is defined by a localized milieu of metabolic inflammation and oxidative stress (171). This reinforces that standard prognostic markers are context-dependent and must be interpreted against host metabolic health. Recently, composite indices including neutrophil-to-lymphocyte ratio (NLR), platelet-to-lymphocyte ratio (PLR), lymphocyte-to-monocyte ratio (LMR), systemic inflammation response index (SIRI), and pan-immune-inflammation value (PIV), have been used to stratify prognosis across colorectal, hepatocellular, gastric, and head and neck cancers, although body composition appears to shift their predictive value and optimal cut points, and further research is needed (172–175). Cytokine and gene-expression signatures add resolution: lower on-treatment IL-6 and IL-8 levels are associated with favorable outcomes following immune checkpoint blockade (176), and a four-gene inflammatory signature and T cell exhaustion signature associate with nivolumab response in HCC (177). Together, these findings highlight the divergent immune states that accompany variation in ICI outcomes (177).

Although the optimal biomarker combination remains undefined, several measures appear particularly informative. A practical core panel could integrate CT- or MRI-derived visceral adiposity and dual-energy X-ray absorptiometry (DXA)-derived measures of body composition with markers of insulin resistance and glycemic control, including fasting insulin or C-peptide, glucose, and HbA1c; adipose dysfunction, particularly leptin, adiponectin, and their ratio; and systemic inflammation, including hsCRP and IL-6. For patients receiving immunotherapy, these objective host measures should be combined with tumor-specific indicators such as the abundance and spatial organization of CD8+ T cells, immunosuppressive myeloid populations, and exhausted or checkpoint-expressing T cells. Serial changes in these measures following weight loss or treatment may ultimately be more informative than single baseline values. Metabolomic, lipidomic, and/or microbiome signatures may also be informative but require prospective validation.

Taken together, these findings support replacing BMI-only risk stratification with parsimonious, multidimensional, and longitudinal phenotypes that integrate objective body composition, adipose tissue inflammation and dysfunction, metabolic health, systemic inflammation, and tumor immune architecture. Immediate priorities are to harmonize measurement methods and clinically meaningful cut points, determine whether candidate markers add predictive value beyond standard clinical variables, and incorporate serial sampling into prospective prevention and treatment trials across diverse populations and tumor types. Validated biomarker panels should distinguish markers of underlying cancer risk and prognosis from those that genuinely predict intervention response, identify reversible biology, and guide the selection and monitoring of lifestyle, metabolic, and immunologic strategies for individual patients.

## 11 Future research priorities

The next research phase for preventing or intercepting obesity-related cancers should move beyond BMI toward biologically informed measures of obesity that incorporate body composition, visceral adiposity, metabolic health, and inflammatory biomarkers. Not all individuals with obesity share the same metabolic or inflammatory phenotype, and not all tissues respond the same to those stresses, so these differences likely contribute to the heterogeneity observed in cancer risk, disease progression, and therapeutic response. Future mechanistic studies should use longitudinal clinical cohorts with integrated molecular and immune profiling to define how obesity-associated tissue remodeling evolves across cancer initiation, progression, metastasis, and treatment response.

Clinical trials evaluating lifestyle interventions, bariatric surgery, GLP-1RAs, and anti-inflammatory therapies should prospectively collect fasting serum or plasma, peripheral blood mononuclear cells, and, when feasible, paired tumor and adjacent adipose tissue at baseline and prespecified follow-up points. Priority biomarkers should include fasting insulin or C-peptide, glucose, HbA1c, leptin, adiponectin and their ratio, hsCRP, IL-6, and lipid measures, together with imaging-derived visceral adiposity and skeletal muscle mass whenever feasible. Immunotherapy trials should additionally assess IL-8; circulating and tumor-associated CD8+ T cell abundance, spatial distribution, activation, cytotoxicity, and exhaustion; PD-L1; immunosuppressive myeloid and macrophage populations; and TREM2-expressing LAM density and indoleamine 2,3-dioxygenase 1 (IDO1) expression. Tissue analyses should quantify CLS, adipocyte hypertrophy, fibrosis, and relevant inflammatory pathways, with metabolomic, lipidomic, and microbiome profiles incorporated, when possible, as exploratory measures. Serial changes in these biomarkers may be more informative than baseline values alone for identifying biological response and predicting cancer prevention, recurrence, or treatment outcomes.

Given obesity is increasingly recognized as an important risk factor for hematological malignancies (2), there are several unanswered questions regarding this understudied link, including i) obesity-driven immune remodeling in bone marrow; ii) interactions between bone marrow adipocytes and malignant hematopoietic cells; iii) biomarkers predicting response to GLP-1 based therapies, as described above; and iv) combination approaches integrating metabolic therapies with immunotherapy in hematologic malignancies.

The obesity-cancer relationship is shaped by structural, environmental, behavioral, biological, and healthcare factors; therefore, health equity must also be prioritized (178). Progress will require close integration of oncology, immunology, metabolism, nutrition, epidemiology, behavioral science, and implementation science. Preclinical models must also better capture the biological complexity and heterogeneity of human obesity, including age, sex, diet composition, weight cycling, metabolic dysfunction, and associated comorbidities. New approach methodologies (NAMs), which originated in regulatory toxicology but are increasingly emphasized in basic and translational research, encompass methods that replace, reduce, or refine animal testing. Complementary approaches, including adipose-tumor-immune co-cultures, patient-derived organoids, tumor spheroids, organ-on-chip, and other microphysiological systems, computational models, and ex vivo tissue models, each address distinct aspects of obesity-associated tumor biology that remain difficult to recapitulate in conventional non-animal experimental systems.

Although not new, *Drosophila melanogaster* represents a whole-organism, alternative approach to traditional vertebrate animal models, and is an underutilized model that bridges reductionist *in vitro* systems and mammalian models for interrogating diet, systemic metabolism, and cancer-related tissue changes within an intact, genetically tractable host (179). Its short life cycle, low cost, conserved insulin, TOR, JAK/STAT, and JNK signaling, and deep genetic toolkit, make it well-suited for rapid mechanistic screening (179, 180). However, because flies lack adaptive immunity, they cannot model T cell-mediated immune surveillance, immune checkpoint biology, or immunotherapy responses that are central to obesity-associated cancer immunity (181). Despite this limitation, as demonstrated by Hirabayashi and colleagues (180), *Drosophila* can nevertheless help prioritize conserved molecular pathways and gene–diet interactions via rapid genetic screening under high sugar diet conditions prior to validation in vertebrate models of diet-induced obesity and cancer.

## 12 Conclusions

Obesity-associated cancer arises from a heterogeneous and potentially reversible host state in which adipose tissue dysfunction, endocrine and metabolic dysregulation, chronic inflammation, and impaired immunity interact with tissue-specific biology, including remodeling of bone marrow. Recognizing obesity in these biological terms creates opportunities to intervene across the cancer continuum through lifestyle, metabolic, and immunomodulatory approaches. The challenge now is to translate this mechanistic understanding into precise strategies that prevent cancer, intercept its progression, improve treatment response, and support long-term survivorship.
